# From brain to worksite: the role of fNIRS in cognitive studies and worker safety

**DOI:** 10.3389/fpubh.2023.1256895

**Published:** 2023-10-25

**Authors:** Yang Han, Jianling Huang, Yang Yin, Huihua Chen

**Affiliations:** School of Civil Engineering, Central South University, Changsha, China

**Keywords:** functional near-infrared spectroscopy, construction safety, construction workers, cognition process, Hazard recognition, decision-making, literature review

## Abstract

Effective hazard recognition and decision-making are crucial factors in ensuring workplace safety in the construction industry. Workers’ cognition closely relates to that hazard-handling behavior. Functional near-infrared spectroscopy (fNIRS) is a neurotechique tool that can evaluate the concentration vibration of oxygenated hemoglobin $\left[HbO_{2}\right]$ and deoxygenated hemoglobin [HbR] to reflect the cognition process. It is essential to monitor workers’ brain activity by fNIRS to analyze their cognitive status and reveal the mechanism in hazard recognition and decision-making process, providing guidance for capability evaluation and management enhancement. This review offers a systematic assessment of fNIRS, encompassing the basic theory, experiment analysis, data analysis, and discussion. A literature search and content analysis are conducted to identify the application of fNIRS in construction safety research, the limitations of selected studies, and the prospects of fNIRS in future research. This article serves as a guide for researchers keen on harnessing fNIRS to bolster construction safety standards and forwards insightful recommendations for subsequent studies.

## 1. Introduction

Dynamic-complex working environment makes the construction industry one of the most dangerous fields. According to the International Labor Organization (ILO), over 381,000 workers worldwide die from occupational injuries each year, with construction workers facing 3–4 times higher fatality rates, which escalate to 4–6 times in developing countries (1). According to the Ministry of Housing and Urban–Rural Development (MHURD), more than 794 Chinese construction workers lost their lives in construction accidents in 2022 (2). In the United States, occupations related to construction and extraction recorded 18.3% of all workplace fatalities in 2021, totaling 951 (3). Consequently, there is an urgent global mandate to enhance construction safety management to mitigate fatal accidents.

Construction projects, characterized by their inherent diversity and complexity, are breeding grounds for numerous workplace hazards such as machinery mishaps, falls, and fires (4). The failure to identify site hazards significantly contributes to unsafe behavior among construction workers, which is implicated in over 70% of construction site accidents (5). Furthermore, research indicates that up to 57% of job site hazards remain unrecognized, exacerbating the risk of accidents (6). Consequently, developing and implementing effective hazard recognition strategies emerge as a critical component of risk management and related safety research aimed at identifying and mitigating various site safety risks more proficiently. Moreover, considering that over 49% of workplace accidents are primarily attributed to the unsafe acts of construction workers (7), it underscores the imperative need to foster the research of safety about informed risk decision-making processes.

Hazard recognition and risk decision-making have garnered substantial attention in the academic sphere, catalyzing a shift toward more objective and scientifically rigorous research methodologies. Traditional research methodologies have relied heavily on surveys, encompassing questionnaires, interviews, and site surveys, to delve into the intrinsic mechanisms (8–10). However, these methods are hampered by subjective and memory biases, and the societal backdrop of safety research often discourages workers from disclosing unsafe behaviors during surveys, interrupting daily operations on sites. To circumvent these limitations, experimental methods have been adopted, where participants engage in risk identification or operational tasks within immersive virtual environments of workplaces, offering a more objective tool for evaluating construction workers’ risk identification and decision-making capabilities (11–16). Despite these advancements, the safety research on a comprehensive understanding of the cognitive mechanisms underlying hazard identification and decision-making among construction workers remains inadequate.

Addressing this gap, the emerging field of neuro-engineering management leverages advancements in sensor technologies to integrate neuroscience research methods into construction safety studies. This interdisciplinary approach facilitates the exploration of human cognition and behavior using advanced neuro-techniques such as electroencephalogram (EEG), functional near-infrared spectroscopy (fNIRS), and eye-tracker (17). EEG and eye-tracker could enhance the studies of brain activities and have been applied in research on hazard recognition and decision-making of construction workers systematically reviewed (18, 19). However, non-direct measurements of the eye tracker and low movement tolerance of EEG restrict the application in cognitive process mechanisms research. These limitations underscore the necessity for a more direct and adaptable tool that can seamlessly integrate into the dynamic environment of construction sites.

**Figure 1:**
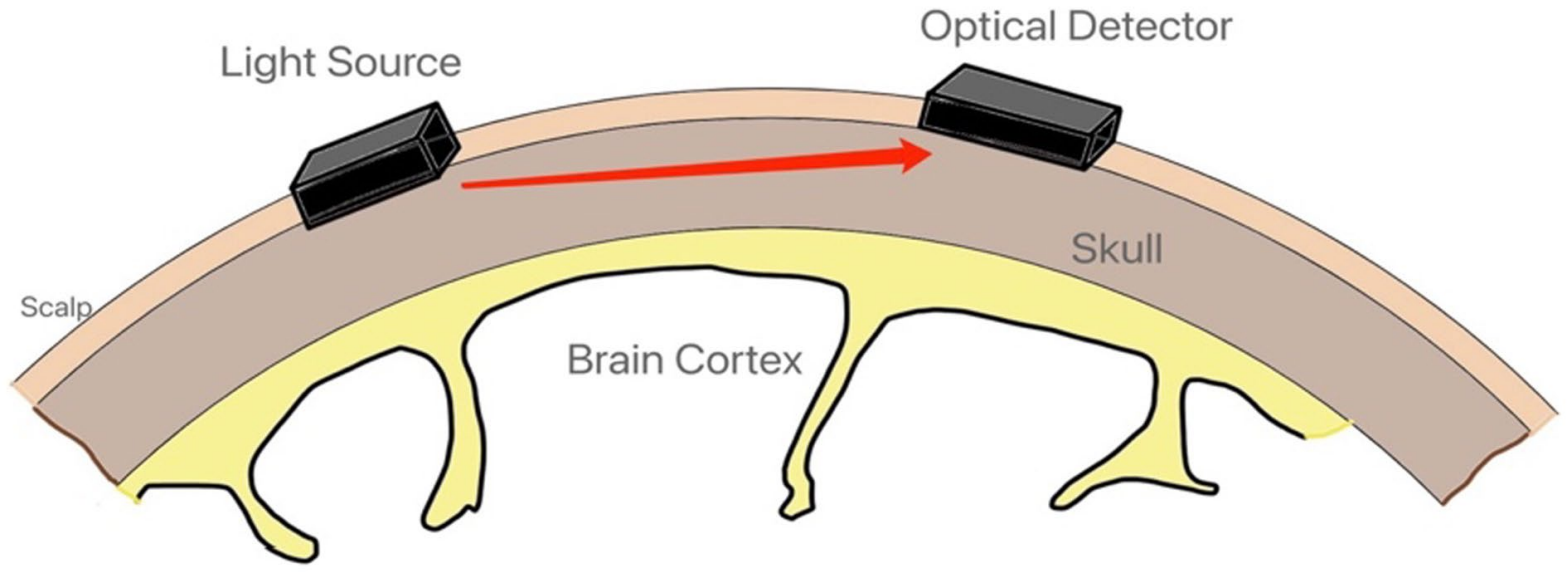
Schematic representation of the basic theory of the fNIRS device.

Here, fNIRS emerges as a pivotal tool, utilizing near-infrared light (~650–1,000 nm) to irradiate the human brain and collect scattered light through an optical detector (Figure 1), thereby directly detecting variations in oxygenated and deoxygenated hemoglobin concentrations in the cortex (20). These variations, supported by cerebral blood flow (CBF), indicate different brain activation states and are central to the cognitive processes involved in hazard recognition and decision-making. The modified Beer–Lambert law facilitates the statistical analysis of these concentration changes (ΔHbO2 and ΔHbR), offering an objective response to the associated CBF dynamics (21). The advent of miniaturized, wearable fNIRS devices has further propelled the field, enabling wireless cognition detection with high movement tolerance, thereby offering enhanced safety, convenience, and minimal disruption to daily work compared to traditional neuroscience methods (22). This development ensures the authenticity of the data collected and enhances the feasibility of implementing fNIRS technology on a larger scale in the construction industry.

Given these promising attributes, fNIRS has rapidly gained traction as a vital scientific tool in cognitive process research, presenting a promising avenue for unraveling the mechanisms of hazard recognition and decision-making in construction safety (23). Since its initial application in 2018, there has been a burgeoning interest in utilizing fNIRS for construction safety research, showcasing its potential as a burgeoning neuro-management technique in this domain (24). Its ability to provide direct, real-time insights into the cognitive processes underlying hazard recognition and decision-making by detecting hemoglobin concentration variation in the brain regions reflecting the cognition status with high movement tolerance and positions it as a revolutionary tool in advancing construction safety research.

However, the current landscape is marred by a lack of systematic reviews, limiting the full realization of fNIRS’s potential in construction safety research. There is an urgent need to scrutinize existing publications to furnish scholars venturing into fNIRS-based construction safety research with organized guidance on research topics, methodologies, and analysis of findings. This endeavor aims to foster collaborative knowledge sharing and research expansion, offering constructive recommendations for future scholarly explorations and management practices in construction safety through a comprehensive examination of pertinent studies in this arena.

## 2. Research methodology

**Figure 2:**
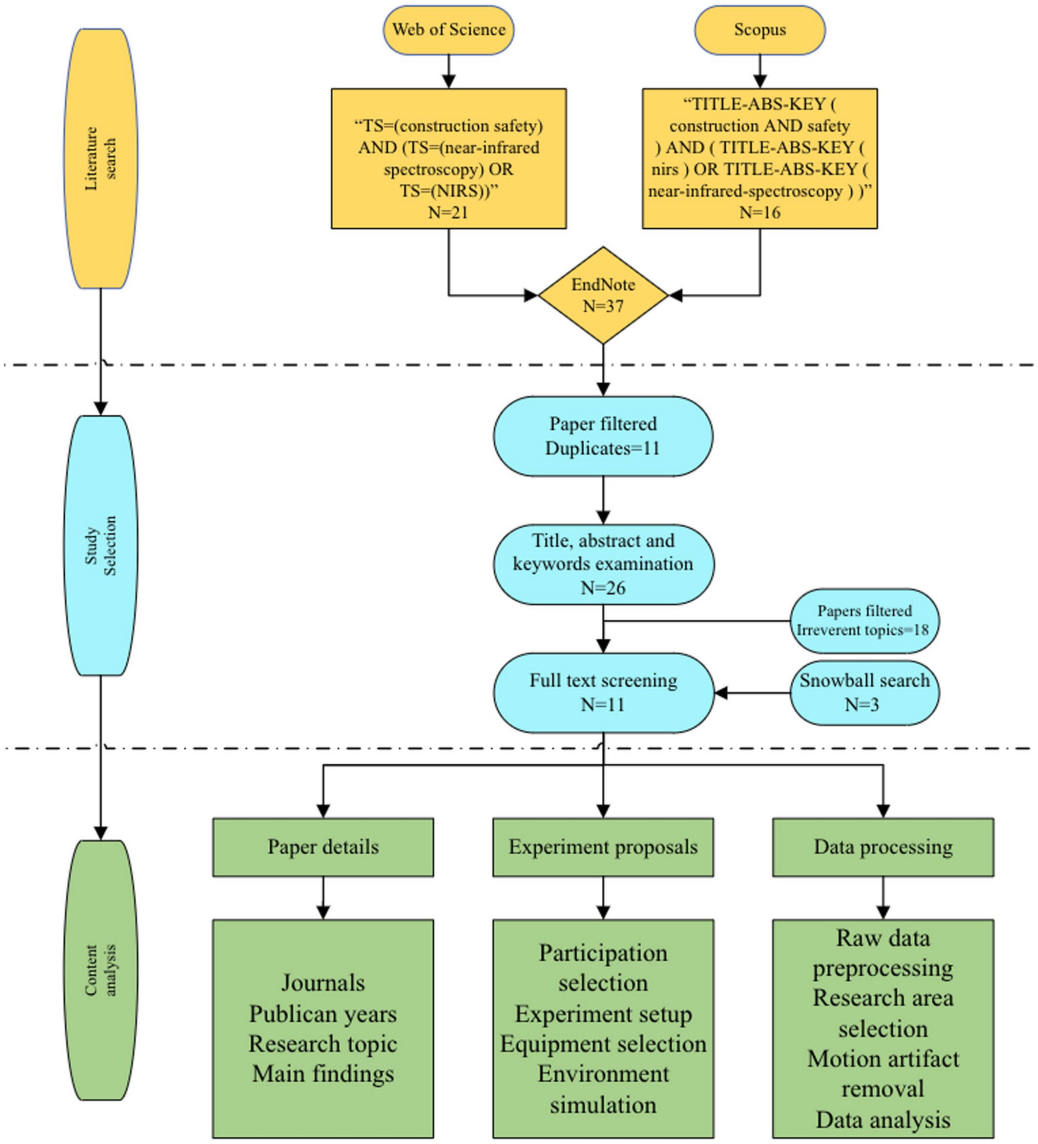
Research methodology.

This study proposes a rigorous three-step methodology to facilitate a comprehensive literature review (Figure 2). Initially, two preeminent academic databases are utilized to procure articles pertinent to this investigation. Subsequently, the identified academic findings undergo a meticulous two-stage screening process. During this phase, papers failing to satisfy the predetermined criteria are excluded from further consideration, thereby delineating the boundaries of this literature review. Finally, a thorough examination of the full texts of the selected articles is undertaken to ascertain the research objectives, experimental methodologies, and data analysis techniques employed.

The Web of Science and Scopus are two of the most reputable scientific databases, encompassing over 15,000 scientific journals globally. An exhaustive search is conducted within these databases, scrutinizing literature titles, abstracts, and keywords to identify relevant academic publications. The focal point of this literature review is the application of functional near-infrared spectroscopy (fNIRS) in enhancing safety within the construction sector, with a particular emphasis on utilizing NIRS to monitor the cognitive states or mental workload of construction workers, thereby fostering a safer work environment. The research scope is bifurcated into two primary dimensions: near-infrared spectroscopy and construction safety. Consequently, the search queries formulated for the databases above are as follows: for Web of Science, “TS = (construction safety) AND (TS = (near-infrared spectroscopy) OR TS = (NIRS)),” and for Scopus, “TITLE-ABS-KEY (construction AND safety) AND (TITLE-ABS-KEY (nirs) OR TITLE-ABS-KEY (near-infrared-spectroscopy)).” In these queries, the pivotal research terms are linked using the “AND” operator, while the critical term “near-infrared spectroscopy” is coupled with its acronym “NIRS” using the “OR” operator.

A further screening process is instituted to ascertain the relevance and alignment of the articles retrieved with the research objectives, guided by pre-established criteria. Firstly, the literature selection is confined to English language publications, given that most seminal works and high-quality information in this domain are predominantly published in English. Secondly, the articles must explicitly employ near-infrared spectroscopy to assess the cognitive dynamics of individuals engaged in construction-related tasks, thereby aligning with the dual focus on near-infrared spectroscopy and construction safety. Lastly, considering the developing integration of near-infrared spectroscopy in construction safety research, this study encompasses peer-reviewed articles, review papers, and conference proceedings to provide a holistic overview of the current research landscape.

Articles failing to meet the stipulated criteria are excluded from further analysis. Upon the completion of the database search, a three-tiered approach is adopted to refine the selection: (1) Preliminary screening through an analysis of titles, abstracts, and keywords to eliminate duplicates and irrelevant literature, adhering to the established screening guidelines. (2) Detailed full-text review to identify articles that fulfill the research criteria for in-depth analysis. (3) Implementation of a snowball search strategy to explore the citations of the articles shortlisted during the second stage, with the aim of incorporating additional literature that, although not identified in the initial database search, aligns with the study’s screening principles for a comprehensive analysis.

A systematic search was carried out using predetermined key terms on Web of Science and Scopus. This resulted in the retrieval of 21 articles from Web of Science and 16 from Scopus. Out of the 37 articles identified, 11 were found to be duplicates and were subsequently excluded. A further 18 articles were removed due to their lack of relevance to the research topic. A supplementary snowball search recommended the addition of three more publications. After the selection process, 11 articles were finalized for this review.

## 3. Experiment analysis

This section explores key considerations in applying functional Near-Infrared Spectroscopy (fNIRS) to construction safety research, encompassing detection area identification, equipment selection, foundational theory comprehension, task design, and participant selection. It delves into how fNIRS is employed to measure brain activity in different regions, with a focus on the prefrontal cortex involved in hazard recognition and decision-making in construction safety.

### 3.1. Detection area

**Figure 3:**
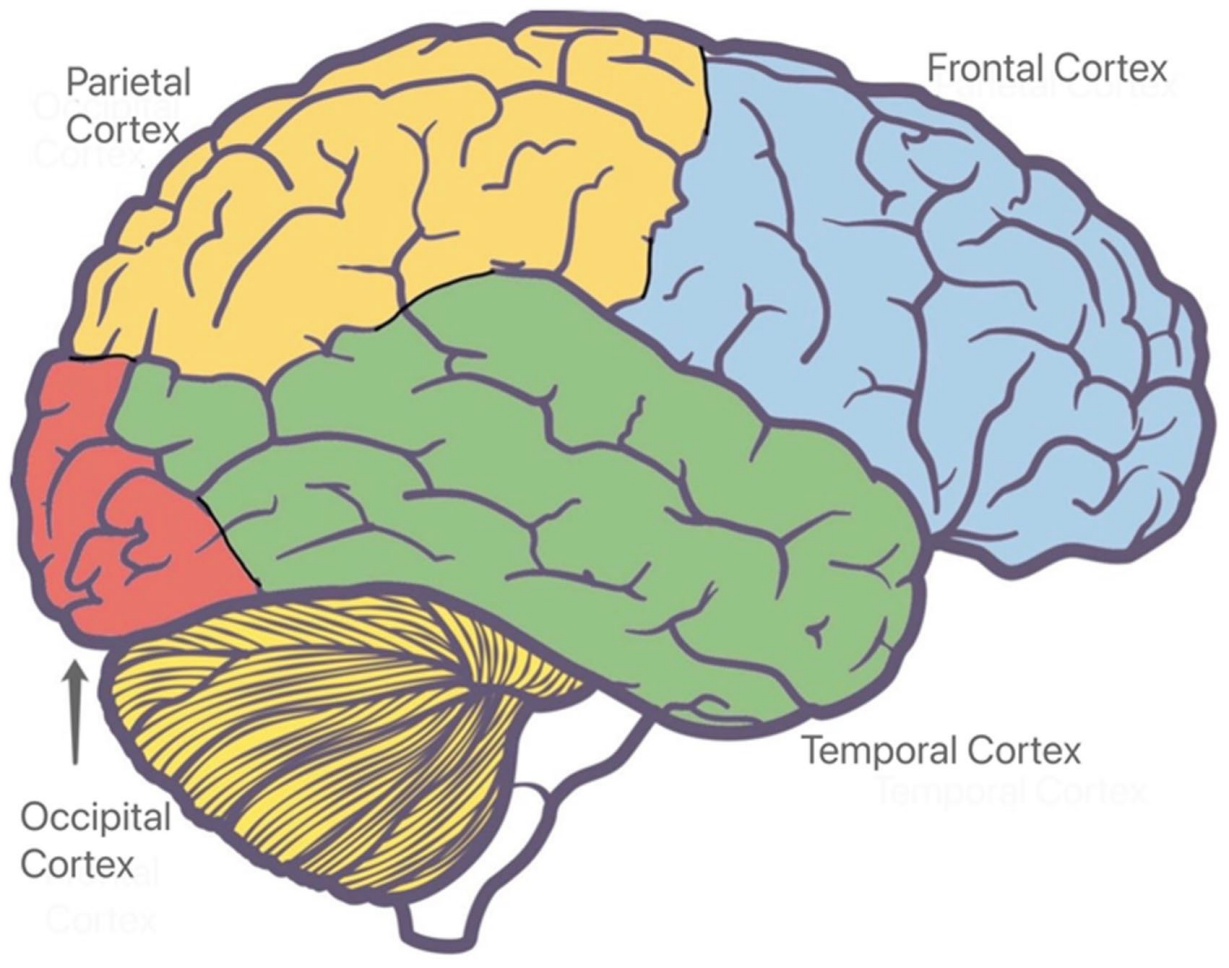
Brain area in fNIRS-based construction safety research.

Brain regions are associated with various specific functions, and fNIRS can be applied to confirm the brain active state of different regions by estimating data variables such as hemoglobin concentration (25). Therefore, identifying the brain regions for measurement is a crucial component of fNIRS-based studies. The human brain consists of four parts in the fNIRS-based construction safety research, such as: the frontal, parietal, temporal, and occipital lobes (Figure 3).

Within the frontal lobe, the blue area depicted in Figure 3 represents the prefrontal cortex (PFC), which is closely associated with functions such as thinking, planning, management, and motor execution (26). Previous studies focusing on the PFC have attempted to identify subjects’ emotional regulation and mental stress (27, 28). Since the PFC is involved in brain functions necessary for hazard recognition and decision-making by workers, existing fNIRS-based studies have predominantly chosen the PFC as the detection area to explore the cognitive mechanism of hazard recognition and decision-making in construction safety management (29, 30).

### 3.2. Apparatus

#### 3.2.1. Equipment evolution

**Figure 4:**
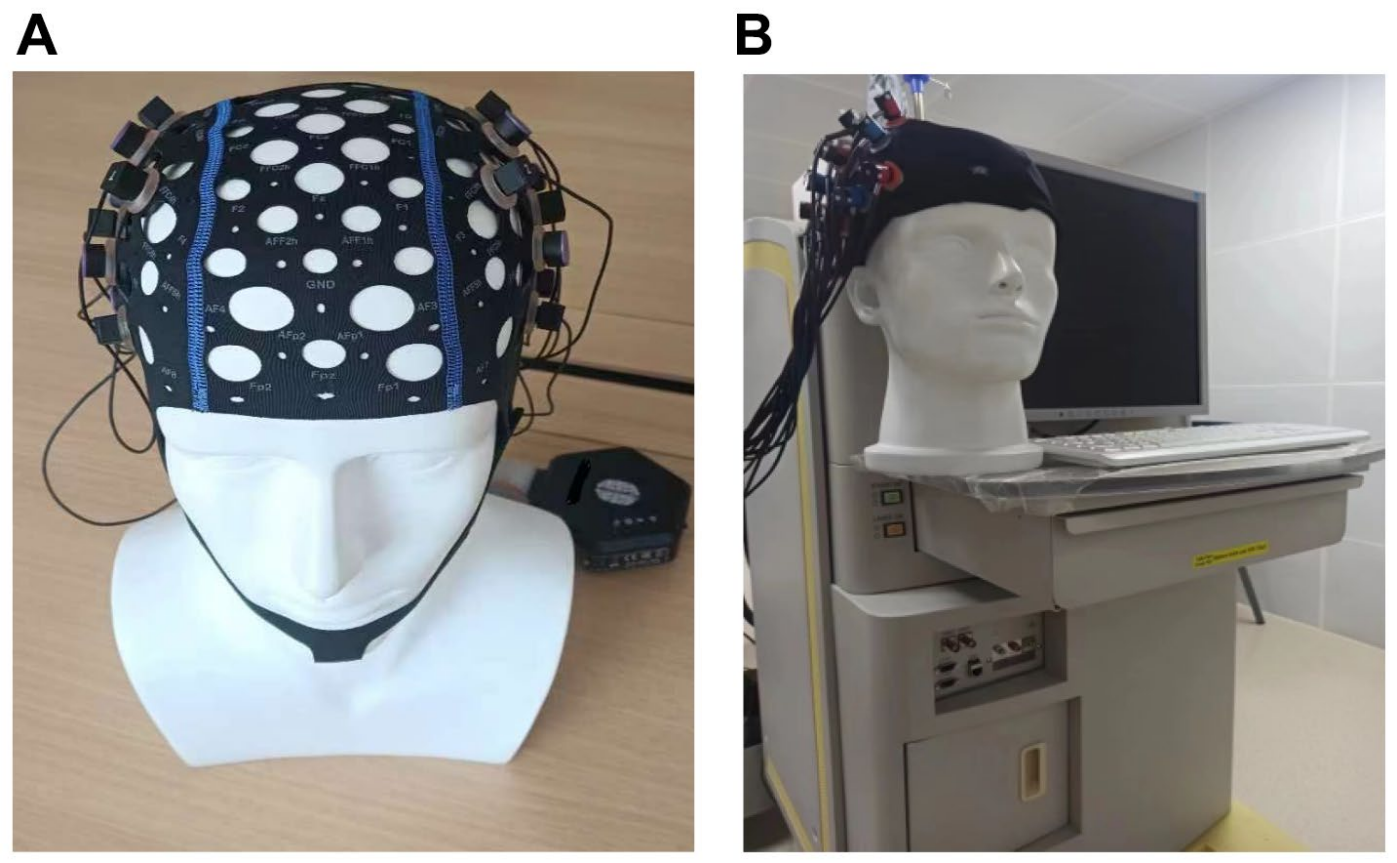
**(A)** Wearable and **(B)** indoor fNIRS devices.

In fNIRS-based research, equipment selection is a critical factor that influences experimental operation, test cost, and measurement quality (Figure 4). Early functional near-infrared spectroscopy devices were bulky and primarily used for indoor experiments (31). However, the advent of wearable fNIRS devices has garnered increased attention from scholars, particularly those interested in applying the technique to construction safety management research (32).

To ensure sufficient fNIRS detection, researchers should consider their research objectives when determining the spatial resolution, sampling rate, and number of channels for choosing fNIRS devices. Although higher resolution devices offer more precise localization of brain activity, they tend to be more expensive and require more sophisticated analysis techniques. Similarly, while higher sampling rates provide more detailed temporal information, they may generate larger datasets and demand more processing power. Additionally, including more detection channels can lead to better coverage and localization, but may also increase the system’s cost and complexity. For example, to detect PFC and motor cortex of human brain, the selected fNIRS device-Oxymon (33) has 24 channels (34).

After considering the factors mentioned above, researchers should investigate whether the fNIRS device includes compatible data analysis software or if additional software is required to process and analyze the collected data. If the experiment involves combining fNIRS with other neuroimaging techniques (e.g., EEG, MRI), it is essential to ensure that the selected fNIRS device is compatible with the other equipment and allows for synchronized data collection. For instance, when integrating an eye-tracker, Liao et al. (29) choose NirSmart (35) with 20 channels.

#### 3.2.2. Multimodal insights

Furthermore, with the development of neuroscience, multimodal study has been proposed as a new research agenda. To carry out joint research with different human cognition indicators associated, a multimodal monitoring method has been generated by integrating several neuro techniques, such as eye tracking, EEG, and fNIRS (36). In the fNIRS-based experiment research of construction safety, scholars typically choose eye tracking for multimodal monitoring (29). Besides, even carrying out experiments in simulated construction scenarios, the participant injuries cannot be avoided entirely. Thus, researchers tried to conduct the fNIRS-based study in a mix-reality environment with a virtual technique like VR, in which participants can explore the surrounding information or perform interactive actions realistically and safely (37). Furthermore, the model development of VR scenarios should achieve more realistic with various construction hazards, which would be a challenge for future fNIRS-based research.

### 3.3. Task design

The design of fNIRS-based experiments in construction safety typically focuses on hazard recognition and decision-making. Participants are tasked with identifying hazards or completing practical operational tasks in realistic environments that reflect the daily work of construction workers.

#### 3.3.1. Hazard recognition

The hazard recognition process is a fundamental aspect of safety activity, which involves signal detection, comprehension, and projection (38). This process requires the involvement of cognitive resources and includes the tasks of detection, evaluation, and forecasting. The brain cognition principle indicates that the increase in cerebral activity leads to the regional blood flow variation for delivering glucose and oxygen for metabolic demands (23). This neural phenomenon is explicitly reflected as the value vibration of indicators: $\left[HbO_{2}\right]$ and [HbR] (39). Typically, the representation of brain activities is considered to be the concentration changes of oxygenated hemoglobin ΔHbO2 (40–42). For instance, in transportation neuroscience research, it has been reported that measuring ΔHbO2 in the PFC effectively captures brain cortex activities, serving as a highly sensitive indicator of CBF (43). Through information synthesizing, the prefrontal cortex coordinates the act and thought based on internal goals via the nerve signal transmission within the brain system during hazard recognition (23, 44). In conclusion, by measuring the oxygenated hemoglobin of PCF, the neuroimaging technology fNIRS offers the methodology to quantitatively describe cognitive activities in the hazard recognition process (24).

In studies utilizing fNIRS for risk identification, some researchers focus on specific areas within a single category of risk, such as fatigue (45), risk attitude (46), and experience (47). Other studies have focused on replicating environments with potential hazards to investigate their effects, including settings that mimic workplaces (29), driving scenarios (48), and equipment usage (34). When it comes to construction safety, researchers place greater emphasis on adequately reflecting construction-related risks in their experiments, such as gravitational, electrical, mechanical, chemical, and individual factors.

Early fNIRS-based studies in construction safety primarily emphasized straightforward risk recognition tasks. Participants were presented with 24 images showcasing varied types and levels of risks (24). Throughout these experiments, scholars captured the participants’ BOLD data from the prefrontal cortex using fNIRS. However, the experimental design did not accurately represent the participants’ real-world work situations. Consequently, there were suggestions to deploy fNIRS measurements in more authentic contexts. For instance, controlled experiments were proposed to examine the impact of fatigue on risk identification, specifically within the driving domain (43) and neurorehabilitation (49). Nevertheless, in the realm of construction safety, workers are often extensively engaged in their routine tasks, leaving little to no opportunity for *in situ* fNIRS experimental manipulations (50).

In response to the constraints observed in earlier fNIRS research methodologies, there’s been a notable shift toward lab-simulated experiments. These experiments allow participants to recognize potential construction-related hazards under the monitoring of fNIRS. The setup often involves a predefined route within a lab-created simulation of a construction environment. Participants, wearing fNIRS devices, follow this route and use tools, such as laser pointers, to identify and report perceived risks. Sun and Liao (30) set a precedent in this direction, tailoring their simulated jobsite settings based on expert guidance. In their study, participants were tasked with recognizing hazards over a span of 25 min, from initial spotting to final decision-making. Essential data points like cognitive responses, identified hazards, and time taken were meticulously captured. This structured 25-min time frame was also incorporated by Zhou et al. (51). Their experimental design encompassed multiple phases: preparation, device calibration, task execution, and hazard verification. Deviating from this model, Qingwen Zhang et al. (52) conducted hazard-searching experiments without adhering to the 25-minute time constraint, but before initiating their core experiment, they evaluated participants on their familiarity with the site, understanding of safety protocols, and risk tolerance.

In essence, these fNIRS-simulated studies underscore the advantages of laboratory experiments, notably the ability to maintain controlled conditions while negating participants’ exposure to real-world hazards. However, a tangible disparity remains: the controlled simulations, no matter how intricate, still cannot fully emulate the complexities and nuances of actual construction worksites.

#### 3.3.2. Decision-making

In the realm of fNIRS-based decision-making research, Wilde (53) introduced the risk homeostasis theory in psychology. This theory posits that workers are often inclined to compromise their fundamental interests, such as safety, in pursuit of potential profits amidst risks. Such a mindset might account for why construction site accidents are intrinsically linked to workers engaging in unsafe behaviors (7). Numerous variables come into play in workplace risk decision-making, encompassing factors like time pressure, cognitive demands, and risk attitude. A notable observation is that workers inclined toward unsafe actions tend to focus more on the enticing positive outcomes associated with these risky behaviors (54). Therefore, a comprehensive analysis of these influential variables is indispensable for effective construction safety management.

fNIRS, a non-invasive neuroscience technique, stands out for its wearability and tolerance to movement, making it apt for monitoring workers’ cognitive activities during decision-making processes in construction. This method evaluates changes in the cerebral cortex’s blood oxygen concentration, a direct indicator of brain activity. These changes can shed light on the effects of various influencing factors on cognition. As the intensity of cognitive tasks rises, there’s a commensurate increase in oxygen consumption by the brain’s neurons. This, in turn, leads to variations in the concentration of hemoglobin within the active neural regions, offering insights into cognitive processes (55).

Within the domain of cognitive studies centered on decision-making, control experiments play a pivotal role (56). However, when the focus shifts to decision-making in construction safety, the research dynamics change considerably. The inherently risky and unpredictable nature of construction environments necessitates a more tailored and rigorous experimental design. The aim is to accurately capture the nuanced cognitive processes influenced by factors such as time constraints. Recognizing this specificity, there has been a discernible trend toward adopting simulation experiments, particularly those leveraging the capabilities of fNIRS, in cognitive research related to construction safety. Illustratively, Pooladvand et al. (46) recommended a laboratory-based electrical construction work experiment with mixed reality environment using fNIRS. Within this framework, participants don VR and fNIRS devices to perform high-risk tasks, post-self-assessment, in two scenarios: standard and high-risk with potential rewards, with their behaviors and cognitive states being rigorously recorded. To ensure the simulation’s authenticity, researchers implemented a preliminary questionnaire, and, after a 30-min training, participants began tasks with the fNIRS system recording real-time cognitive data in an immersive environment (37).

### 3.4. Participants selection

The selection of participants can significantly impact the quality of an experiment. For instance, differences in memory performance among subjects can affect fNIRS measuring results (34). To address this issue, scholars recommend administering a brief questionnaire, such as the factor-referenced cognitive test, to ensure that abnormal mental conditions do not affect fNIRS measuring results (57). Additionally, having a sufficient sample size is essential in experimental design to reduce fNIRS measurement errors resulting from individual variations. In the reviewed studies on fNIRS applied in construction safety, the experiment participants range from 14 to 48, with a mean number of 40 and a median of 47. To enhance the reliability of results, researchers suggest having no fewer than 50 participants in psychological experiments, allowing for the simple comparison of two cognitive process indicators with 80% power (58). This assertion is based on the acceptable effect size of *d* = 0.4, with the sample size growing to 100 or more in the presence of within-group variable interactions. The existing sample sizes in studies are less than the ideal estimation, which may impact the reliability of experiment conclusions considering the complicated cognition status and related jumbled and violated measuring data in labor’s daily work.

In the reviewed papers that used experimental research methods with fNIRS, students in the field of civil engineering were selected as participants, with an average age of no less than 20 years (59, 60). High labor costs may be a reason for using students for experiments in some regions, such as the United States (46). Additionally, complex neuroscientific experiment tasks of fNIRS require participants to have a higher ability to learn and understand. However, construction workers are often perceived to lack higher education and to be older, which is contrary to the requirements of the fNIRS-based experiment. No studies have selected construction workers as subjects yet to generate convincing fNIRS experiment results. Moreover, there are fewer personal characteristic differences among students, which has the advantage of controlling irrelevant variables in fNIRS-based experiments (61). However, replacing construction workers with college students as fNIRS experiment participants has aroused suspicion for lacking sufficient working experience in construction. Furthermore, these fNIRS studies do not provide a reasonable explanation for why the findings of undergraduate participating experiments are equally applicable to construction workers, requiring further exploration.

## 4. Data analysis

### 4.1. Feature extraction

Hematoxylin plays an essential role in oxygen transport (62), particularly in industries like construction that involve significant physical work. The brain activity of construction workers leads to an increase in oxygen consumption, causing regional fluctuations in the concentration of oxyhemoglobin ($\left[HbO_{2}\right]$) and deoxyhemoglobin ([HbR]) (63). This forms the basis for fNIRS-based studies on construction safety, which enables the acquisition of $\left[HbO_{2}\right]$ and [HbR] data (64). From this data, it is possible to generate the total hemoglobin concentration ([tHb], i.e., the sum of $\left[HbO_{2}\right]$ and [HbR]) and tissue oxygen saturation ($\left[StO_{2}\right]$) (21). The result of $\left[HbO_{2}\right]$ and [HbR] do not originate from direct fNIRS-based measurements but are calculated by processing the raw light intensity data based on the optical attenuation of the measured near-infrared spectroscopy light (65, 66). However, it is important to note that fNIRS measurements are susceptible to motion artifacts and physiological disturbances, especially when monitoring construction workers. Therefore, the successful extraction of essential features through the processing of raw functional near-infrared spectroscopy data becomes a critical factor in related neuroscience research (67).

In the fNIRS data analysis, the MBLL (Modified Beer–Lambert law) can be used to calculate the optical density variation for high dispersion media, which is the physics underlying the calculation of $\left[HbO_{2}\right]$ and [HbR] (21). The MBLL is generally formulated as (68):

$$\Delta OD=-\log\left[\frac{I}{I^{0}}\;\right]≃\Delta \mu_{\alpha }BL$$

(1)In this equation, ΔOD represents the change in optical density concerning the position of the light source, the position of the fNIRS device probe, the detection light wavelength (λ), and the detection time (t) (66). On the right-hand side of this equation, I denotes the photon flux received by the probe at its position at time t, when the light source position (69). And the incident photon flux of the light source at the same time is then expressed as $I^{0}$. In this equation, $\Delta \mu_{\alpha }$ represents the change in the absorption coefficient (70). B and L shown above are parameters related to the position of the probe and the light source (B is the differential path coefficient, and L is the distance between the probe and the light source) (71). For the calculation of the $\Delta \mu_{\alpha }$ coefficient on the right side of Equation (1), based on the underlying assumption that the optical density variation in fNIRS-based studies is mainly from $\left[HbO_{2}\right]$ and [HbR] (72).

### 4.2. Signal preprocessing

The signal data collected in the reviewed fNIRS-based construction safety research are derived from three components, namely, (i) evoked neurovascular coupling from external stimuli or task design, (ii) spontaneous neurovascular coupling, and (iii) systemic physiological processes, including “physiological disturbances” or “systemic disturbances,” evoked by non-neurovascular coupling. Relying on the fNIRS signal, changes in blood oxygen concentration in the grey matter of the construction worker’s brain can be measured, which also reflects physiologically based systematic confounding in the superficial layers of the head (skin and frontal bone) (73). Recording a systematically confounded fNIRS signal can obscure participants’ brain activity and produce experimental error (74). To ensure effective and accurate statistical analysis of fNIRS-based construction safety research, it is necessary to preprocess signal data to exclude systematic confusion and remove unwanted signals (75).

The usual elements of signal processing and analysis of near-infrared spectroscopy include correction for motion artifacts, short-range correction, and physiological noise separation (66). In experimental studies applying fNIRS, low-pass filtering is one of the most commonly used signal processing methods to reduce experimental error by removing non-evoked signal content (76). As fNIRS-based construction safety research has evolved, data processing methods have been iteratively upgraded. Scholars raise a new approach for motion artifact removal by machine learning which shows a similar treatment effect between walking and stationary state in fNIRS measurement (77).

### 4.3. Analysis method

#### 4.3.1. Statistical models

In the nascent stages of fNIRS-based construction safety research, the primary focus was on the rudimentary analysis of variations in hemoglobin oxygen content, which were then integrated with time series to formulate visualized results for analytical reference (24). However, as the field advanced, the complexity of experimental objectives and procedures increased, necessitating the integration of more sophisticated statistical approaches.

To address this growing complexity, researchers have expanded their analytical toolkit to include a diverse range of statistical models in fNIRS-based studies such as the two-sample *t*-test, paired *t*-test, one-way ANOVA, and multinomial ANOVA (20). Initially, simple *t*-tests were predominantly utilized to analyze mean value differences, offering a straightforward approach to data analysis (78). Subsequently, introducing more complex statistical models became imperative to cater to the evolving needs of fNIRS research. These later developments included the adoption of paired *t*-tests, which have proven instrumental in comparing activation levels under varying brain conditions (79), and the integration of ANOVA models, facilitating a nuanced analysis of data derived from fNIRS-based experiments (80).

Meanwhile, these statistical models reveal distinct advantages and disadvantages in fNIRS experiments. For instance, while *t*-tests are relatively simple to implement and adept at comparing means between two groups, researchers should consider whether the data in fNIRS-based study presuppose a normal distribution (47). Furthermore, the application of *t*-tests in multiple comparisons elevates the risk of type I error, potentially compromising the validity of the research findings for construction safety (81). Conversely, ANOVA models, though capable of handling multiple comparisons simultaneously in fNIRS experiments and offering a holistic view of the data, necessitating larger sample sizes in construction safety research can sometimes be a limiting factor (82). Moreover, interpreting results from multi-factorial ANOVA models can be complex, requiring a profound understanding of the statistical principles and proposing rational experimental designs that underpin these models in fNIRS-based safety research of construction workers (66).

Selecting an appropriate statistical model in the context of fNIRS-based construction safety research is multifaceted and influenced by numerous critical factors (83). These factors encompass specific research objectives, the nature of the collected data, and the intricacies inherent in the experimental design. For instance, in the pursuit of introducing a quantitative metric for assessing the hazard recognition capability of construction workers, researchers strategically opted for multivariate analysis of variance to compare diverse multimodal data, which included fNIRS measurements (30). This selection process demands a profound understanding of the underlying data distribution, considerations regarding sample size, and a meticulous evaluation of the potential consequences associated with both type I and type II errors in fNIRS experiments (84). Consequently, some research teams expanded the cohort of participants involved in fNIRS experiment and augmented the number of measurement channels for fNIRS to accumulate a more extensive dataset, thereby enhancing the robustness of their construction safety analysis (59). Furthermore, in selecting statistical models for the analysis of fNIRS data, it is crucial that these models not only facilitate the extraction of meaningful insights but also contribute to a deeper understanding of the intricate dynamics within the realm of construction safety. As an illustrative example, the adoption of a general linear model to investigate the relationship between individual risk attitudes and decision-making related to construction risk emerged as a rational and well-founded choice (46). Looking toward the future, as fNIRS experiments in construction safety evolve toward greater complexity and comprehensiveness, there is a burgeoning anticipation for the development of specialized statistical tools and software (85). These tools are poised to enable a more exhaustive and intricate analysis of fNIRS data, fostering a deeper and more nuanced understanding of the intricacies within the field of construction safety research.

#### 4.3.2. Machine learning models

Increasing requirements of the fNIRS application in sectors such as healthcare are impeded by restricted monitoring regions (86). This has propelled the evolution of fNIRS devices, like the bundled optode approach, which could potentially augment the number of detection channels by thousands for experiments in construction safety research, thus introducing more complex statistical demands (87). Moreover, statistical analysis of a larger array of fNIRS data features elevates the volume and intricacy of data processing and comparison in construction safety research. These features include peak, mean, skewness, variance, slope, root mean square, and median (88). In response to this research trend, machine learning has been raised to achieve automatic feature extraction and classification tasks, like linear discriminant analysis (LDA), support vector machines (SVM), deep belief networks (DBN), and convolutional neural networks (CNN) (89, 90). Ho et al. (91) suggest a methodology using CNN and DBN achieving better classification accuracies of 84.26 ± 9.10% in comparison of [$HbO_{2}$] and [HbR] concentrations among two mental stages. In the mental load research by fNIRS, researchers compare the effect of a machine learning algorithm in data classification tasks, and it shows that DBN and CNN have higher accuracy than traditional methods like AdaBoost and SVM, one of the most popular models utilized widely for the cognition research of workers (92).

Integrating many algorithms, called ensemble learning, shows the potential for greater classification performance than applying a single traditional model in fNIRS-based research (93). This promising prospect has prompted scholars to consider the application of ensemble learning to fNIRS-based construction safety research, specifically in the cognitive exploration of construction workers. Two major categories of algorithms in ensemble learning contain bagging methods and boosting methods, such as XGBoost and Random Forest (RF). Linear support vector machine and linear discriminant analysis are integrated for classification work to improve the functional performance of fNIRS-based brain-computer interfaces (fNIRS-BCIs) (94). Li et al. (95) report that the ensemble learning of the 2-layer-GA-SVM model could enhance the average accuracy from 70.6 to 84.4% compared to the single GA-SVM model in fNIRS statistical analysis. Li et al. (96) also achieve an effective recognition rate of 94.4% (151/160) by fNIRS data using the gradient boosting tree (GBDT) and random forest (RF) models. These research cases in machine learning aiding data processing in the fNIRS-based study of construction safety are insufficient for further review analysis. Still, it provides guidance and insight for related research and deserves further observation in the future.

## 5. Discussion

### 5.1. Neurotechniques comparison

Functional neuroimaging technologies are pivotal tools in cognitive research, and within this realm, Functional Magnetic Resonance Imaging (fMRI), Electroencephalography (EEG), Electrocardiography (ECG), and Functional Near-Infrared Spectroscopy (fNIRS) are prominent methods (97–99). These technologies possess unique characteristics that make them suitable for cognitive research and application.

For example, fMRI is widely used to capture brain activity by monitoring changes in blood flow and oxygenation levels in specific brain regions (100). Its high spatial resolution allows for precise localization of brain activity, a valuable asset in cognitive research. However, fMRI has limitations, including the large, expensive equipment and the requirement for subjects to remain immobile within a narrow MRI machine, which can be restrictive in cognitive research related to construction worker safety (101).

On the other hand, EEG offers a different perspective on cognitive research (102). It reflects the macroscopic activity of the brain cortex and shares common attributes with fNIRS, such as wearability, wireless functionality, miniaturization, and lightweight design (103, 104). EEG records electrical activity on the scalp, providing insights into brain function in cognitive research. EEG devices are portable, lightweight, and relatively cost-effective compared to fMRI, making them well-suited for monitoring brain activity during various tasks, including cognitive assessments relevant to construction worker safety (105). However, EEG comes with certain limitations. For instance, in construction safety research, participants are required to apply a conductive paste to their scalp. This ensures a stable connection with the EEG electrodes, but it also adds to the overall cost of the experiments (106). Moreover, the nature of a construction worker’s job, which involves large-scale movements and vigorous physical activities, can introduce significant measurement noise into the EEG recordings. This not only impacts the detection precision but also increases the complexity of data processing and interpretation (18).

ECG, another technique in cognitive research, measures the electrical activity of the heart, offering insights into physiological responses during cognitive tasks of workers (107). ECG monitoring is relatively non-invasive, involving the placement of electrodes on the skin, and is well-suited for studying physiological responses in contexts related to construction worker safety. While ECG provides valuable data on cardiovascular responses, it is not a direct measure of brain activity like EEG or fNIRS in construction safety research.

In contrast, fNIRS emerges as a favorable option for investigating construction worker safety. fNIRS technology, due to its wearability, light weight, and tolerance for subject movement, proves ideal for assessing cognitive and physiological responses in dynamic work environments. Unlike EEG and ECG, fNIRS does not require conductive gel, thus eliminating discomfort and simplifying setup procedures (108). Moreover, fNIRS provides a more direct measure of localized changes in brain oxygenation, offering more profound insights into cognitive processes relevant to construction safety tasks. Furthermore, fNIRS exhibits a higher degree of movement tolerance, particularly in operational studies, as participants can wear the fNIRS equipment directly on their heads, providing more comfort compared to fMRI and EEG in construction safety research (109). This heightened tolerance reduces the likelihood of sweat-induced errors in device detection, minimizes exercise-related or physiological artifacts, and decreases the subsequent data processing workload, which is more common in EEG and ECG (18). Consequently, fNIRS demonstrates considerable potential for applications involving collecting long-time series data, specifically in the context of construction worker safety.

Given the dynamic, complex, and cognitively demanding nature of construction work, fNIRS emerges as the more suitable neuroscientific research tool for investigating construction safety. Its portability, comfort, and movement tolerance make it a practical choice for studying the cognitive and physiological responses of construction workers, ultimately contributing to the enhancement of safety measures and training protocols within the construction industry.

### 5.2. Implications

To achieve effective safety management in construction, it is essential to understand the cognitive processes of construction workers. As their work is performed in a complex and hazardous environment, it demands enormous cognitive effort. Therefore, accurate measurement and evaluation approaches are crucial for cognition research in construction safety. Traditional subjective questionnaire methodology is inadequate to capture the dynamic and complex nature of this research. The development of consumer-grade wearable fNIRS devices provides a more objective and accurate method to monitor brain activity in construction workers. In this review research, we have demonstrated the capability of fNIRS to measure cognitive processes and its future potential for multimodal studies. These findings suggest that fNIRS is a valuable tool for measuring the cognitive processes of construction workers on site, ultimately improving safety management in the industry.

Degrees of brain activity is closely related to the vibration of oxyhemoglobin concentration, which can intuitively reflect human cognitive and psychological status more intuitively and provide more accurate data to support experimental studies through fNIRS measurements (23). The success of fNIRS-based experiments requires a sufficient number of participants who receive appropriate pre-task guidance and are adequately representative of the target population. Meanwhile, the experiment design of construction safety research should thoroughly consider the authenticity of the operational environment and the possibility of influence on the measurement data, for which virtual reality has been applied in fNIRS-based studies (46). The fNIRS data can be evaluated to measure the correlation with mental representation, physiological indices, and external factors in workplaces (30). In cognition monitoring, fNIRS measurements produce fewer data noise and motion artifacts, resulting in less error in experimental results and higher data quality. After pre-processing (e.g., low-pass filtering), statistical analysis of fNIRS data can be calculated by the software, in which machine learning demonstrates higher computational power and accuracy in cognition status classification and computation.

According to previous research, fNIRS has demonstrated several advantages in the cognition research of construction workers compared to other measuring methods. Firstly, the portable and wearable nature of fNIRS devices allows for high motion tolerance and use in diverse research environments (104). Meanwhile, fNIRS also has good subject comfort. Secondly, fNIRS is a neuroscience technique that directly detects blood oxygen concentration variables in the cerebral cortex using near-infrared light, allowing for the most direct indication of cognitive activities through calculated [$HbO_{2}$] and [HbR] vibration values. This generated data enables thorough research into the phenomenon of brain blood flow to explore the mechanisms of related cognitive processes. Lastly, fNIRS measurement is time dynamic, recording the [$HbO_{2}$] and [HbR] changes owing to blood flow with the high temporal and spatial resolution, which is compatible with the rapid change and small-time scale of cognitive activity.

The use of fNIRS-based research in construction safety has filled a gap in the neuroscience field and provided a new technical tool for studying human cognitive conditions, which can improve our understanding of hazard recognition and decision-making processes (29). The fNIRS method facilitates an objective assessment of workers’ hazard recognition and decision-making abilities, which can help managers to improve safety training strategies and measure training effectiveness. With the aid of fNIRS-based measurement metrics, managers can provide individualized, customized training programs for construction workers, and future intelligent risk identification capability assessment training systems can be developed. Furthermore, a better understanding of decision-making mechanisms through fNIRS-based research provides an objective basis for improving management methods and identifying the key factors affecting management effectiveness.

### 5.3. Limitation

The fNIRS-based research in construction safety is still in its infancy, and the lack of sufficient study cases limits this present review. Although applying neuroscience techniques in the computation of the cognition process is an emerging research direction, the fNIRS-based study provides a broad view of construction workers’ neuroscience exploration. However, several limitations of fNIRS-based construction safety research expected to be addressed are discussed below:

1. Although researchers have tried to simulate the working environment of construction in a lab with appropriate space and set up risk points based on the actual situation (52), it is still challenging to fully represent the complex environment of actual construction sites (29). The dynamic changes of the construction workplace and the interferences between workers have not been discussed enough and are reflected in the existing research experiments. Moreover, the hazard search tasks designed for fNIRS-based research are of limited difficulty and may not be representative of actual construction workplaces. Furthermore, the sample subjects selected for simulation experiments are mainly young, college-educated males, which differ significantly from the educational profile of construction workers and may introduce bias into the fNIRS-based experiment results (110). Additionally, the ratio of female and older workers among construction workers is constantly vibrating, and these situations have not received sufficient attention in the existing fNIRS-based experimental designs (111). Furthermore, the fNIRS-based literature selected for this review suffers from a small sample size, which may limit the generalizability of the findings.
2. Scholars have focused on time and peak features of $\left[HbO_{2}\right]$ and [HbR] concentration data to evaluate the impact of designed variables on hazard recognition and decision-making through the fNIRS-based experiment with construction workers (24). However, statistical analysis of more fNIRS data features has not been involved in exploring the cognitions status of construction workers, such as skewness, variance, slope, root means square, and median (88). Additionally, the analysis of designed impact variables to hazard recognition and decision-making only considers relevance in the fNIRS-based experiment, and further inquiry into the mechanisms of influence is needed.
3. Scholars have primarily focused on a limited number of indicators to evaluate the cognitive processes of construction workers in hazard recognition and decision-making. However, the cognitive process of construction workers is influenced by various internal and external factors, which are currently underrepresented in experimental analysis. For example, the hazard searching task in fNIRS-based experiments analyzing the implications of risk type on workers’ hazard recognition only considers four main risk events of workplaces, which differs significantly from the actual situation (59). While fNIRS-based experiments have been widely used in various industries to detect workers’ cognitive states, such as mental load, fatigue, and emotional state, the factors that affect workers’ cognition and may lead to unsafe behavior or ignorance of risk are complex (89, 112, 113). However, in the context of construction safety, the current application of this neuroscience technology has mainly focused on the importance of hazard identification and decision-making without exploring the potential impact of other cognitive factors on worker safety. In addition, the statistical analysis stage does not sufficiently consider the joint influence of multiple designed variables on the hazard recognition process for construction workers. Further experimental exploration is needed to investigate the mechanisms of interaction within the influencing factors, the synergistic impact mechanisms on hazard recognition, and the mechanisms of cognitive state formation under the influence in fNIRS-based research.
4. Researchers have primarily focused on exploring the role of different prefrontal cortex (PFC) areas in hazard identification and decision-making in fNIRS-based studies of construction workers (51). However, different brain regions typically work together to perform a specific recognition task, and it is, therefore, worth exploring whether other brain areas of construction workers are involved in the hazard recognition task, as well as their mechanism of action and degree of activation (114). Additionally, due to inadequate spatial resolution, fNIRS is insufficient for exploring cognitive functions in deep brain regions, and multimodal neuro-technique solutions are expected in the future. Furthermore, machine learning algorithms are well-suited for processing large amounts of data and have promising applications in the intelligent analysis of fNIRS data, particularly for feature learning and deep learning for classification. However, there is a lack of participation in machine learning methodology in fNIRS-based cognitive process computation of construction workers, and further research in this area is warranted.

### 5.4. Future direction

To demonstrate the potential of the fNIRS-based study in construction safety, the following recommendations for future research should be considered:

1. To demonstrate the potential of fNIRS-based studies in construction safety, it is recommended to improve the experimental design by simulating the working conditions of construction workers more comprehensively, including the full range of potential hazards. Researchers should select representative subjects with adequate sample sizes and properties compositions of gender and age that reflect the actual situation of construction workers in fNIRS-based cognitive experiments. Simplified experimental designs should be avoided, and tasks should correspond to the actual working conditions of construction workers, evaluating the influences of collaborative working among workers. In addition, incorporating immersive and realistic experimental environments through VR technology can enhance the fNIRS-based experiment design in the laboratory.
2. Scholars should consider incorporating additional data features into their statistical analysis. In addition to the time and peak features currently being used, other features such as skewness, variance, slope, root means square, and median should also be explored. This will allow for a more comprehensive understanding of the patterns and trends within the fNIRS data, which can provide valuable insights into the cognitive processes of construction workers. To support this effort, researchers should draw upon theoretical and experimental studies in neuroscience to identify relevant data features and develop appropriate statistical models.
3. In the domain of future fNIRS-based research aimed at enhancing the safety of construction workers, several crucial avenues for investigation emerge. Firstly, researchers should endeavor to expand the scope of influencing factors integrated into their fNIRS-based experiments. These factors should encompass a comprehensive array of variables in construction safety, including environmental conditions, psychological states, individual variances, and related elements. Incorporating these multifaceted variables will facilitate the acquisition of a more comprehensive understanding of the cognitive processes involved in recognizing hazards and decision-making among construction workers. Such a broader perspective will yield more precise insights conducive to enhancing safety management. Furthermore, it is essential to prioritize the examination of the mechanisms that underlie the interaction among these influencing factors. Understanding how internal and external elements synergistically affect cognitive processes will effectively bridge the knowledge gap about forming cognitive states in construction workers. This, in turn, will lead to a better understanding of construction workers’ cognitive processes within safety environments and support the development of more effective safety strategies. In addition, fNIRS-based research can investigate the cognitive foundations of hazard recognition and decision-making. This entails an exploration of cognitive variations across diverse work environments, analyzing workers’ physiological responses to hazard cues, and investigating the neural substrates of decision-making processes. These research endeavors will shed light on the cognitive challenges construction workers encounter in hazardous contexts, consequently informing the design of more effective safety training and management protocols. Ultimately, these research proposals have the potential to enhance the safety of construction workers significantly. Through rigorous cognitive investigations, we can attain a more profound comprehension of the pivotal factors influencing hazard recognition and decision-making, thereby enabling enhanced training and support, mitigating accident risks, and elevating overall safety standards at construction sites.
4. To improve the effectiveness of fNIRS-based research in construction safety, it is necessary to increase the number of brain regions explored in experiments. Researchers should consider the mechanisms by which different brain regions function in cognitive processes. To address this issue, fNIRS can be integrated with other neuroscience techniques, such as EEG, eye tracking, and fMRI, to conduct multimodal studies in construction safety. Multimodal research can compensate for the lack of spatial measurements in fNIRS devices and provide more comprehensive data to support cognitive studies of construction workers. Furthermore, the generated large amount of data from multimodal research can be analyzed and processed by artificial intelligent algorithms such as machine learning. Meanwhile, a suite of intelligent data analysis systems consisting of deep learning methods can be expected as a promising direction for future fNIRS-based research, such as: convolutional neural networks (CNN), recurrent neural networks (RNN) and graphical neural networks (GNN).

## 6. Conclusion

The fNIRS-based research provides a valuable methodology to enhance the understanding of the cognitive processes of workers in construction safety for both academics and managers. This review provided a systematic analysis of the fNIRS-based application in exploring construction workers’ cognitive process of hazard recognition and decision-making. Scholars have used fNIRS to study the influence of multiple variables on cognitive processes in hazard cognition and decision-making by computing concentration changes in [$HbO_{2}$] and [HbR]. Moreover, the review outlined the basic theory and significant highlights and issues in fNIRS-based experimental design (detection area, participant selection, experimental device, and task design) and data analysis (data extraction, signal processing, computation method, and objective analysis).

This study identified gaps in the current fNIRS-based research and provided suggestions for future exploration. The design in the existing fNIRS-based experimental studies did not sufficiently simulate the realities of construction workers’ daily work, and researchers need to investigate the mechanisms of workers’ cognitive processes further. Multimodal approaches, such as integrating fNIRS with other neuroscience techniques, show promise for advancing the exploration of cognitive processes. Additionally, machine learning algorithms can be used to process and analyze large amounts of data generated by multimodal studies.

Therefore, future fNIRS-based exploration in construction safety should focus on theoretical development, experimental design, and algorithmic applications. This review provides guidance for new researchers in fNIRS-based safety research on the cognitive processes of construction workers, which would benefit construction management improvements.

## Author contributions

YH: Conceptualization, Data curation, Formal analysis, Investigation, Methodology, Project administration, Writing – original draft, Writing – review & editing. JH: Conceptualization, Formal analysis, Methodology, Project administration, Supervision, Validation, Writing – review & editing. YY: Data curation, Formal analysis, Investigation, Methodology, Writing – review & editing. HC: Conceptualization, Formal analysis, Project administration, Supervision, Validation, Writing – review & editing.
